# Caries prevalence and dental health of 8–12 year-old children in Damascus city in Syria during the Syrian Crisis; a cross-sectional epidemiological oral health survey

**DOI:** 10.1186/s12903-019-0713-9

**Published:** 2019-01-15

**Authors:** Muhammed Al-Huda Ballouk, Mayssoon Dashash

**Affiliations:** 1 Paediatric Dentistry Department, Faculty of Dentistry, Damascus University, Damascus, Syria; 2 Paediatric Dentistry Department, Faculty of Dentistry, Damascus University, Damascus, Syria; 3 Centre for Measurement & Evaluation in Higher Education, Ministry of Higher Education, Damascus, Syria

**Keywords:** Caries, Oral health, Prevalence, DMFT, Children, Syria, Damascus

## Abstract

**Background:**

There was an immense need for studies evaluating the dental health status in Syrian children, especially under the current circumstances of the Syrian crisis. No contemporary data was available. The aim of this study was to assess the current dental health status in children aged 8 to 12 years in Damascus city.

**Methods:**

The study was a cross-sectional epidemiological school-based oral health survey using stratified random cluster sampling. A total of 1500 children were clinically examined. For each child, personal information together with DMFT and dmft indices were recorded. Statistical analysis was undertaken to investigate the effects of different factors on caries prevalence. ANOVA, and Chi Square tests were both utilised.

**Results:**

The caries prevalence for the whole city was at (79.1%). The mean DMFT was (2.03 ± 1.81) and the mean dmft was (2.47 ± 2.94). Of the DMFT index mean value, (91.14%) was for decayed and missing permanent teeth. Of the dmft index mean value, (89.1%) was for decayed and missing deciduous teeth. The most affected teeth were the permanent first molars (1.58 ± 1.51). There is a statistically significant relationship between the used indices means and the children’s distribution as to the city’s localities.

**Conclusions:**

Dental caries was higher than expected. Health promotion programmes are essential and of critical importance in order to improve the dental health status.

## Background

Oral health is considered as a very important indicator that can be employed to indicate the general health status for persons [1]. It also positively adds up to the person’s physical mental and social health [2, 3]. A good oral health during childhood contributes to a better oral health as an adult later on [4, 5]. Knowing the current oral health status in one society has a big role as well as impact on any therapeutic or preventive interventions which can be adopted on a large scale whether in the educational curricula and teaching strategies or even in oral health awareness programmes [6, 7]. It is also known that the oral health well-being in children is both an important issue and a high responsibility for the society as a whole [8, 9]. A good oral health in one person means mainly the good status of the teeth, gingiva and the supporting periodontal tissues in this person [10].

Therefore, dental health is a fundamental important aspect of the person’s general health. The presence of dental caries is a great indicator for the later development of many oral health related diseases that cause pain and sometimes impairment, and this is besides being the first cause for most teeth losses worldwide [10].

The aim of this study was to assess the current status of the dental health in children aged 8 to 12 years in Damascus city and to help enabling health-care professionals to improve the oral health status amongst the whole population.

## Methods

### Study design and approach

The study was a cross-sectional epidemiological school-based oral health survey and was conducted in Damascus city in Syria. The field practical part of the study was conducted from September 2016 to January 2017. The study design and methods took into consideration the World Health Organisation’s (WHO) guidelines published as the “Oral Health Surveys; Basic Methods” in its 5th edition [11]. Damascus city was divided, as to the health sectors map taken from the Ministry of Health, into 9 localities (Al-Zahera & Midan sector, the Old City sector, Al-Mazzeh sector, Al-Muhajirin sector, Dummar & its surroundings sector, Kafarsoseh sector, Barzeh & Rekn Al-Dien sector, Kossor & Kassaa sector, Malki & Abu Rumaneh sector).

### Ethical approval and official permissions

Ethical Approval was obtained from the Ethics Committee and the Board of Scientific Research at the Faculty of Dentistry at Damascus University. Official written permissions were also obtained from the authority bodies (Ministry of Health, Ministry of Education, Ministry of Higher Education and Research, and the School Authorities). Also, informed consent was obtained from children’s parents/carers in order to recruit their children in this study.

### The study sample and sampling method

The sample size was estimated at a precision level of (0.05) to be (1278). Still, the precision level was raised to (0.03) to increase the study’s quality and reliability making the sample size reach (1444) children. The number was rounded up to (1500) for easier calculations. The confidence interval was at (95%). The multi-stage cluster sampling method was used to select the sample objects. The study sample was comprised of (1500) children of which (745) were females (49.7%) and (755) were males (50.3%). The schools were randomly selected from the lists of schools provided by the Ministry of Education for each locality.

### Inclusion criteria

Children aged 8 to 12 years old and residing in Damascus city in the time of the study.

Having no personal reasons that prevent the children from participating (whether for the children themselves or their carers).

### Exclusion criteria

Children having any active infectious diseases or any medical conditions that suggest special extra precautions for the children’s own safety and health as well as for the examiner’s.

### The examination procedure

All children were examined by a single examiner (MAB). Personal information, DMFT and dmft indices were recorded as a whole and in details for each tooth as well. In addition, the participating children were taught how to brush their teeth well and were given oral hygiene instructions to improve their overall oral health. Contact was made with the parents/carers of those children in need for treatments and they were referred to the nearest dental office or dental care centre. The diagnostic kit used for the examination included oral mirrors, dental explorers, dental tweezers, and periodontal probes. Also, a head-held light was used. The children were examined at their schools.

### Statistical processing

The raw data was processed using IBM SPSS Statistics version 23. Descriptive statistics study was done to reach the mean values for the used indices. Moreover, ANOVA, and Chi Square tests were implemented to study the relationships between the indices means and the distribution according to the localities.

## Results

*Caries prevalence rate* for the whole city was (79.1%). The highest caries prevalence rate in the localities was seen in Dummar sector reaching (90.4%) while the lowest was recorded in Barzeh and Rekn Al-Dien sector and it reached (66.7%). *The mean DMFT* for the whole city was (2.03 ± 1.81). The highest DMFT mean in the localities was seen in Al-Mazzeh sector reaching (2.58 ± 2.25) while the lowest was recorded in Barzeh and Rekn Al-Dien sector and it reached (0.93 ± 1.34). *The mean dmft* for the whole city was (2.47 ± 2.94). The highest dmf mean in the localities was seen in Dummar sector reaching (4.71 ± 2.95) while the lowest was recorded in Al-Mazzeh sector and it reached (0.80 ± 1.68).

*The ft out of the dmft percentage* was (10.9%), and *the FT out of the DMFT percentage* was (8.86%), both too low. In other words, over (90%) of both the DMFT and dmft indices mean values combined were for decayed and missing teeth. *The teeth with the most lesions* were the first permanent molars, with a mean of decayed first permanent molars of (1.58 ± 1.51) for the whole city. The highest mean for the decayed permanent first molars in the localities was seen in Dummar sector reaching (2.08 ± 1.66) while the lowest was recorded in Barzeh and Rekn Al-Dien sector and it reached (0.84 ± 1.24). The mean of missing first permanent molars for the whole city was (0.13 ± 0.74).

Table 2 shows the statistical significance study for caries prevalence rates in relationship to the distribution as to the localities while Table 3 shows the statistical significance study for the DMFT and dmft indices means in relationship to the distribution as to the localities.

## Discussion

### Possible reasons for findings and justification of area selection

Malnutrition, the increased consumption of carbohydrates and added artificial sugars found in cheap and low-quality foods, having no water fluoridation, the absence of many primary health-care services, the bad financial situation for plenty, and the crisis situation by itself (which further helped in causing all the aforementioned problems), are all factors which have negatively attributed to the oral health situation (dental health status included) for the whole population. With such factors taking effect, it is extremely important to know the ground situation we are standing on before implementing any large-scale interventions or adopting nationwide strategies. For these reasons Damascus city was chosen as a location for our research. Damascus now has got residents from various other destinations all around Syria. Many of them fled from their home towns to Damascus because of the war, which makes Damascus a city that has got the full spectrum of the Syrian population not only for being the capital but also for having somehow better situations. Therefore, we can say that our conclusions and results are truly representative of the children’s dental health in Damascus and can be a good indicator as well for the children’s dental situation in Syria generally speaking.

### Geo-demographical comparisons

Table 1 shows a comprehensive comparison amongst research results for many countries and including caries prevalence rates, DMFT, and dmft, as well as the age groups targeted. The studies selected were research results published in journals that are indexed by Medline and searchable through PubMed and report DMFT or dmft indices results. This table covers the literature that was published until 10 years from carrying out the present study.

**Table 1:** Caries prevalence rates and the mean dmft/DMFT indices’ values in recent related literature, chronologically

Publication Date	First Author	Research Date	Country	Caries Prevalence	dmft	Age Group (for dmft)	DMFT	Age Group (for DMFT)	Ref.
2017	Al Mashhadani	2013	Dubai (UAE)	About 60%	3.87	5–6	1.83	12–15	[16]
2016	Al-Thani	2015	Qatar	71.4%	4.2	6	–	–	[17]
2015	Kumar	2014	India	47.2%	–	–	4.82	12–15	[18]
2015	Zhang	2011–2012	Yunnan Province (China)	40%	–	–	0.9	12	[19]
2014	Salman	2013	Lattakia (Syria)	67.1%	–	–	2.35	13–15	[20]
2014	Gao	2012	Shaanxi (Western China)	67% (4–6) 24% (12–15)	3.05	4–6	0.45	12–15	[21]
2013	Besseling	2011	Laos	85.4%	8.15	5–7	1.44	11–12	[22]
2012	Dawani	2011	Sadr Town (Pakistan)	51%	2.08	3–6	–	–	[23]
2012	Dashash	2009	Damascus (Syria)	61%	3.27	5	–	–	[24]
2012	Chu	2009	Hong Kong	49%	2.2	5	–	–	[25]
2012	Monse	2006	Philippines	97% (6) 82% (12)	8.4	6	2.9	12	[26]
2010	Gökalp	2004–2005	Turkey	69.8% (5) 61.1% (12) 66.4% (15)	3.7	5	1.9 2.3	12 15	[27]
2010	Al-Haddad	2002–2003	Sana’a (Yemen)	96%	4.16	6–14	2.25	6–14	[28]
2010	El-Nadeef	2001–2002	UAE	83%	5.1	5	–	–	[12]
2009	Al Hindawi	2008	Eastern Sector of (Syria)	80.4%	3.13	6–7	0.05	6–7	[29]
2009	Nurelhuda	2007–2008	Khartoum State (Sudan)	30.5%	–	–	0.42	12	[30]
2009	Phelan	2007	NSW (Australia)	About 35%	1.53	5–6	0.74	11–12	[31]
2009	Jürgensen	2006	Laos	56%	–	–	1.8	12	[32]
2007	Salman	2006	Lattakia (Syria)	88.47%	–	–	2.83	13–15	[33]
2007	Batwala	2006	Uganda	About 27%	2.7	5–12	1.5	5–12	[34]
2006	Kalyvas	2001	Attica County (Greece)	48.4%	2.6	5	–	–	[35]

Despite the fact that the biggest share of these variations could be due to the differences in ethnicities, regions, environments, the age groups studied, time of the survey, and various other conditions, but it is also worth mentioning that plenty of studies proved that the presence of dental caries in the primary dentition is a very important predictor for having caries in the mixed and permanent dentitions. The same way it goes for the mixed dentition being also a predictor for the permanent dentition [12–15]. Therefore, it was justified comparing the caries prevalence rates of one research in any population to the other international and local literature regardless of age, race, regions, and so on. As for the teeth status, the indices used were the decayed, missing, or filled indices for both the permanent and deciduous teeth rather than the decayed, ex-foliated, or filled teeth index. The fact that enabled reaching accurate measurements for both indices (the DMFT and the dmft) which are comparable to most of the international and local literature again regardless of age, race, regions, and any other factors.

Of the DMFT index mean value, (91.14%) was for decayed and missing permanent teeth. Of the dmft index mean value, (89.1%) was for decayed and missing deciduous teeth. In other words, the ft mean was equal to only (10.9%) of the dmft mean and the FT mean was equal to (8.86%) of the DMFT mean. These percentages are too low and could be due to the lack of knowledge regarding the necessity and importance of treating children’s teeth. Also, it may be the result of the bad financial situation leaving no ability for the families to cover the dental treatment costs.

The teeth with the most lesions were the first permanent molars, with a mean of decayed first permanent molars of (1.58) for the whole city. On the other hand, the mean of missing first permanent molars for the whole city was (0.13). These high numbers indicate the ignorance of parents regarding the importance of the first permanent molars as well as the absence of any preventive measures like fluoride varnish and pits and fissures sealants. Moreover, it could be the reason of the parents not knowing that these teeth in particular are permanent teeth and the most important of all teeth as well, and possibly mistaking them for deciduous teeth. Also, it could be the result of the widespread misconception that primary teeth are of no importance, which along with mistaking the first permanent molars for primary teeth may have been the main factors that contributed to this problem.

### Relationships outcomes by indices

As shown in Table 2, the *P*-value was 0.000 and therefore it is statistically significant (actually very significant, for it is less than 0.01). So, it can be concluded that there is an assessed relationship between the localities in which the children reside and the caries prevalence. In other words, more attention and special preventive measures should be taken in the areas with the top caries prevalence rates. Also, it can be said that the same way it goes in management as most important first, any therapeutic or preventive nation-wide interventions should target the areas that are most in need first even within the same city.

**Table 2:** Caries prevalence rates in the sample children according to the localities and the statistical study for caries prevalence in relationship to the distribution as to the localities

Sector	Children’s Counts and Percentages					Chi Square Value	*P*-Value* for the Caries Prevalence Rates
	Caries-Free Counts	Caries-Free Percentages	With Caries Counts	With Caries Percentages	Total Counts		
Zahera & Midan	25	12.9	169	87.1	194	67.377740	**0.000** **Significant**
Old City	27	17.4	128	82.6	155		
Al-Mazzeh	32	20.1	127	79.9	159		
Al-Muhajirin	41	27.3	109	72.7	150		
Dummar	19	9.6	178	90.4	197		
Kafarsoseh	47	31.3	103	68.7	150		
Barzeh	51	33.3	102	66.7	153		
Kossor & Kassaa	55	28.8	136	71.2	191		
Malki	17	11.3	134	88.7	151		
Total	314 Children	20.9%	1186 Children	79.1%	1500 Children		

*(Chi Square)

From Table 3, the *P*-values for both indices were 0.000 and signifying the presence of statistical differences and significance (again very significant since they are less than 0.01). Consequently, it can be concluded that there is an assessed relationship between the localities in which the children reside and the means for both indices, the DMFT and the dmft, which also indicates that there is more immense need for extensive preventive interventions in the localities that are with the highest means and hence they should be targeted first as part of any future awareness or interventional programmes.

**Table 3:** The DMFT and dmft indices means in the sample children according to the localities and the statistical study for these means in relationship to the distribution as to the localities

Sector Name	Mean DMFT	SD ±	*P*-Value**	Mean dmft	SD ±	*P*-Value**
Zahera & Midan	2.35	1.71	**.000** **Significant**	4.30	3.66	**.000** **Significant**
Old City	1.68	1.63		2.77	2.99	
Al-Mazzeh	2.58	2.25		0.80	1.68	
Al-Muhajirin	1.89	1.48		1.16	1.93	
Dummar	2.20	1.67		4.71	2.95	
Kafarsoseh	2.65	2.15		0.87	1.56	
Barzeh	0.93	1.34		3.30	2.97	
Kossor	1.64	1.72		1.49	1.89	
Malki	2.30	1.59		1.97	2.31	
Total	2.03	1.81		2.47	2.94	

**(ANOVA)

All participating children were given thorough advice on the best ways to care for their “smiles” and keep good oral health. These good oral hygiene instructions were passed to the children individually when examining each child and also in groups with the help of visual aids and illustrations. Teachers and school supervisors were also requested to pay good attention to the students’ health and hygiene generally and to the oral health in specific. One of the present study’s limitations that should be mentioned in here is not having carried out a test-retest.

## Conclusions

Caries prevalence rates as well as both dmft and DMFT indices means were all high indicating that the dental health has reached a low level. Hence, we can say that the oral health status is also low due to the bad dental situation which in turn requires special care and coordinating all the efforts seeking the better. There is an immense need for comprehensive preventive programmes as well as increasing the awareness about oral health generally and dental health in specific. Any awareness or preventive programmes should target the localities with higher caries levels first.

### Limitations

*Chronological limitations*: The practical field part of the study was conducted in 2017 and 2016. Such studies are a continuous need and therefore it is highly advisable that this study is repeated within a period of 3 to 4 years. *Spatial Limitations*: The study was undertaken in Damascus and there is a necessity for carrying out studies like this present study in other Syrian cities. *Logical Limitations:* It is worth mentioning that no test-retest was carried out and this can be considered a limitation for the study. However, repeating such an examination procedure could have caused the children group to be re-examined some irritation and disturbance and could have affected their cooperation negatively in the dental clinic later on even slightly.

## Abbreviations

DMFT: Decayed, missing, and filled permanent teeth
dmft: Decayed, missing, and filled temporary teeth
MAB: Muhammed Al-Huda BALLOUK
SD: Standard deviation
WHO: World Health Organisation
